# A review on effects of conjugated linoleic fatty acid (CLA) upon body composition and energetic metabolism

**DOI:** 10.1186/s12970-015-0097-4

**Published:** 2015-09-17

**Authors:** Tatiana Ederich Lehnen, Marcondes Ramos da Silva, Augusto Camacho, Aline Marcadenti, Alexandre Machado Lehnen

**Affiliations:** Faculdade Sogipa de Educação Física, Porto Alegre, Brazil; Instituto de Cardiologia/Fundação Universitária de Cardiologia (IC/FUC), Porto Alegre, Brazil; Universidade Federal de Ciências da Saúde de Porto Alegre (UFCSPA), Porto Alegre, Brazil; Instituto de Cardiologia do Rio Grande do Sul, Av. Princesa Isabel, 395 Santana, 90620-001 Porto Alegre, RS Brazil

## Abstract

Conjugated linoleic acid (CLA) is highly found in fats from ruminants and it appears to favorably modify the body composition and cardiometabolic risk factors. The capacity of CLA to reduce the body fat levels as well as its benefic actions on glycemic profile, atherosclerosis and cancer has already been proved in experimental models. Furthermore, CLA supplementation may modulate the immune function, help re-synthetize of glycogen and potentiate the bone mineralization. CLA supplementation also could increase the lipolysis and reduce the accumulation of fatty acids on the adipose tissue; the putative mechanisms involved may be its action in reducing the lipase lipoprotein activity and to increase the carnitine-palmitoil-transferase-1 (CAT-1) activity, its interaction with PPARγ, and to raise the expression of UCP-1. Although studies made in human have shown some benefits of CLA supplementation as the weight loss, the results are still discordant. Moreover, some have shown adverse effects, such as negative effects on glucose metabolism and lipid profile. The purpose of this article is to review the available data regarding the benefits of CLA on the energetic metabolism and body composition, emphasizing action mechanisms.

## Introduction

Although many research studies are inconclusive about functional foods, their benefits to health have often been discussed, calling the attention of the scientific community [1–3]. Thus, several studies were performed claiming that functional foods are essential for health and have helped reduce the risk of developing various chronic diseases [4–6]. This functional property concerns the metabolic or physiological role played by the nutrient or non-nutrient in growth, development, maturity and other normal functions of the human organism. However, studies on nutraceutics (foods with a medicinal function) lack further explanation, especially regarding the associated protective effects. The doses indicated generate doubts that these effects will be achieved, and also regarding the possible adverse effects of their long term use [1–3].

Several classes of substance which are naturally present in foods or produced by food technology have functional properties. One of these substances is conjugated linoleic acid (CLA) - a fatty acid which presents a linoleic acid isomer (C18:2, n-6) and has been considered an antiobesity agent, and can be useful in the weight reduction process [7]. Although the initial results were found only in an animal model [8, 9], more recent research on humans suggests that CLA would act to reduce adiposity through modulating properties in the lipid metabolism [10, 11]. However, doubts remain as to the action mechanisms of CLA in adipocytes, leading to the reduction of body fat and, especially, the safety of supplementation of this compound.

Therefore, the purpose of this review is to describe the effect of CLA supplementation on body composition, particularly on the reduction of adiposity, focusing on possible action mechanisms.

## Conjugated linoleic acid

**Fig. 1:**
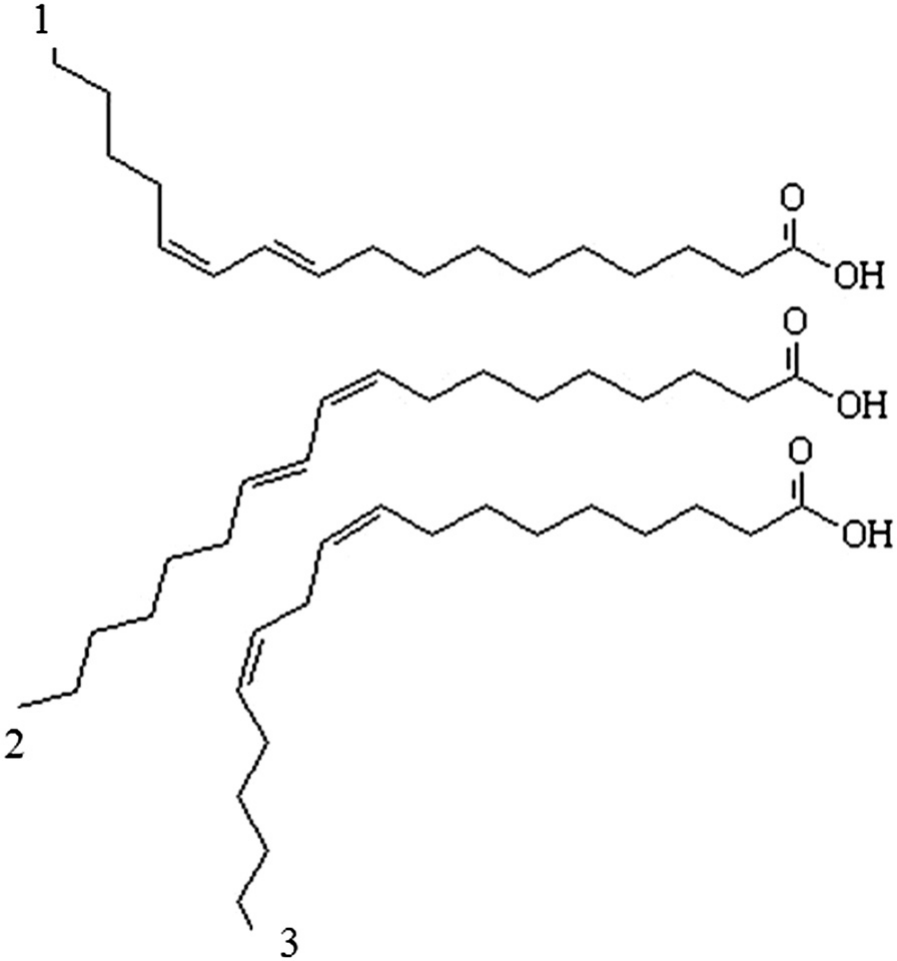
Isomer structure. (1) represents CLA 10-trans and 12-cis; (2) indicate CLA 9-cis and 11-trans; (3) C18:2 9-cis and 12-cis

Conjugated linoleic acid (CLA) is a term that describes a group of fatty acids with 18 atoms of carbon, and the geometric isomers consist of linoleic acid [12]. This is a common name given to a group of position isomers with two double bonds separated by a methylene group [7, 13]. This conjugation of the double bond is generally in positions 9 and 11 or 10 and 12, and may be a *cis* or *trans* configuration (Fig. 1).

CLA is produced naturally in the digestive tract of ruminants such as cattle, goats, sheep, buffalo, and to a lesser degree in pigs, chickens and turkeys, and the synthesis occur due to fermentative bacteria, *Butyrivibrio Fibrisolvens*, which isomerize the linoleic acid in CLA or by synthesis via α9-desaturase of 11-*trans* octadecanoic acid [14]. The fat in beef contains about 1.7 to 10.8 mg CLA/g of fat with 9-*cis* and 11-*trans* isomers. It is also found in dairy products (milks and derivatives) [6].

CLA can be obtained by means of enzyme α9-desaturase which promotes the desaturation of the 11-*trans* octadecanoic acid. Several different isomers of CLA such as 11-*trans* and 9-*cis* are the best known because they are found in food [7, 12]. It is also possible to obtain CLA in an industrial form, through the partial hydrogenation of linoleic acid or by thermal treatments, aiming to produce a compound with maximum biological activity and with a defined chemical composition [10].

CLA has a major role in the lipid metabolism, especially as regards the oxidative cellular system, which explains many physiological properties of fatty acids. Their action on the lipid metabolism is associated with the inhibition of the entry of glucose into the adipocytes, and may lead to changes in the insulin metabolism and cause situations of hyperinsulinemia, as well as the increase of inflammatory markers [15, 16].

There are many investigations to evaluate the influences of CLA on the energetic metabolism, promoting significant changes in the lipid metabolism and in body composition [9–11, 17–20]. As a result, some effects can be cited such as: reduction of body fat, improved insulin resistance, antithrombogenic and anticarcinogenic effects, reduction of atherosclerosis, improved lipid profile, modulation of the immune system and stimulation of bone mineralization, and also reduced blood glucose. The most studied CLA supplementation effect is its capacity to alter the body composition, promoting an increase in lean mass and reduction of the fatty mass.

## Putative mechanisms of action

The possible action mechanisms which show that CLA can alter the body composition involve metabolic changes that favor the reduction of the lipogenesis and the potentiation of lipolysis, accompanied by the oxidation of fatty acid in the skeletal muscle, due to increased carnitine palmitoil-transferase-1 activity and action, or possibly because of adipocyte differentiation inhibition [7]. Therefore, researchers have evaluated the action of CLA supplementation on the lipid and hormone profile, and the activity of the enzymes involved in the oxidation process [21].

Studies have demonstrated that isomers 10-*trans* and 12-*cis*, differently from the 9-*cis* 11-*trans* of CLA, increase lipolysis significantly in the human adipocytes, and also have the function of diminishing the synthesis of fatty acids [15]. This would explain, in part, the possible action mechanisms of CLA on the body composition. Although various studies were i*n vitro*, the metabolic hypotheses to explain the body fat reducing action of CLA began based on control of the expression of genes involved in the differentiation of pre-adipocytes into mature adipocytes, in other words, the expression of these genes would result in reducing lipogenesis [22, 23].

In turn, the peroxisome proliferator-activated receptors (PPARs) are nuclear transcription factors that play a central role in the storage and catabolism of fatty acids (FA). They are part of a class of nuclear receptors that belong to the family of the nuclear receptor of the steroid, retinoid and thyroid receptors. Three isoforms of the nuclear receptor have already been identified, PPARα, PPARβ and PPARγ. PPARα and β are involved in the lipid metabolism (especially the proteins related to FA oxidation) and glucose, and PPARγ is involved in adipocyte differentiation [24, 25].

**Fig. 2:**
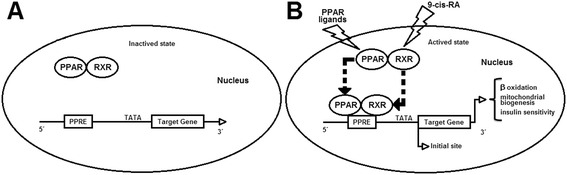
Mechanism for transcriptional activation by PPAR. Panel **a** shows the inactivated state, without gene transcriptional of any target genes. Panel **b** shows the activation of PPAR by PPAR ligands and RXR by 9-cis-RA (9-cis-retinoic acid), thereby stimulating target genes transcription by binding to specific DNA sequence (peroxisome proliferators – PPRE) leading to increased β-oxidation, mitochondrial biogenesis and insulin sensitivity

Figure 2 shows the activation mechanism and requires the release of the co-repressor complex (histone deacetylase activity) by a binder, and the recruitment of the co-activator complex (acetyltransferase activity). The activated PPAR:RxR complex binds to the elements that are responsive to peroxisome proliferators (PPRE), producing changes in the chromatin structure, giving rise to a transcriptionally competent structure. Hence, it seems that the CLA interacts with the Co-activator complex PPAR increasing the gene transcription related to the differentiation of adipocytes, lipolysis (β-oxidation), mitochondrial biogenesis and insulin sensitivity, and collectively, it is related to the weight loss effect [24].

The effects of CLA in lipid and glucose metabolism on body composition are mediated by the activation or inhibition of the PPARs, especially PPARγ. The inhibition of PPARγ by CLA (isomer 10-*trans*, 12-*cis*) leads to the reduction of body fat by modulation of the gene expression in the sense that it inhibits cell differentiation and alters the activity of proteins involved in lipogenesis and in lipolysis [26]. Evidence suggests that the activation of PPARγ can diminish the progression of atherosclerosis and increase sensitivity to insulin, and may be a potential therapeutic target for the treatment of various diseases, including diabetes mellitus type 2 (DM2) and dyslipidemia.

In adipocytes, PPARγ regulates the expression of genes involved in the lipid metabolism, including acyl CoA-synthetase and lipoprotein lipase (LPL). The expression of the transport protein of fatty acids involved in the uptake of lipids by the adipocytes is also controlled by PPARγ [25].

The reduction of body fat occurs not due to the reduction in the number of adipocytes but rather by the reduction of their size. Considering that the size of the adipose cells is directly elated to the triglyceride content inside the cells, its reduction results in a smaller cell size. The increased β-oxidation of mitochondrial fatty acids induced by CLA may be responsible for the reduction of triacylglycerol synthesis, not depositing them in the adipocyte, but reducing their size [8].

The fatty acid is transported into the mitochondria by the carnitine-palmitoil-transferase (CPT) complex. Three enzymatic components are involved: CPT-1, CPT-2 and carnitine acylcarnitine translocase (CATC) [27]. The fatty acids are activated by the acyl-CoA synthetase enzyme forming an activated complex (fatty acyl-CoA), with the carnitine-palmitoil-transferase (CPT-1) enzyme. This complex penetrates the mitochondrial membrane and reaches the intermembrane space. Acyl-CoA is regenerated with the release of carnitine in the CPT-2 reaction. Once it reaches the mitochondrial matrix, the long chain fatty acid (LCFA) is oxidized to generate adenosine triphosphate (ATP) through the β-oxidation of the fatty acids [27]. CLA supplementation would increase the concentration and activity of CPT-1. Thus, collectively, the increased lipolysis, the reduction of lipase lipoprotein activity and increased carnitine-palmitoil-transferase-1 (CAT-1) activity lead to the reduction of the accumulation of fatty acids in the adipose and muscle tissues. These action mechanisms are those most discussed by the researchers [8]. Inside the mitochondria the fatty acids are oxidized by β–oxidation reactions and Cycle of Krebs (CK), releasing the H^+^ and e- which are carried (NADH^+2^ and FADH_2_) to the respiratory chain (1). The gradient of H^+^ and e- between the intermembrane space and the matrix determines its return passing by the ATP synthase protein (2) with a synthesis of ATP (coupled reaction) or by the uncoupling protein (3) producing heat.

However, the rationale that CLA stimulates lipolysis only by increasing CPT-1 is valid (and limited) for situations in which β-oxidation (capacity to generate ATP through the successive break down of fatty acid carbon) is more efficient than the transport of the fatty acyl-CoA complex to the mitochondrial matrix. In this way it is possible and logical to say that CLA supplementation (increasing CPT-1 concentration and activation) would only have a potential effect on physically active individuals, particularly for those whose β-oxidation is more efficient than the transport of fatty acid itself to the mitochondrial matrix.

On the other hand, weight loss with CLA supplementation could be explained by its association with the uncoupling protein of the respiratory chain (UCP), potentiating the β-oxidation capacity [28]. The respiratory chain or electron transport chain is formed by a series of transport compounds located in the inner membrane of the mitochondria. The last of these of these compounds is called cytochrome-oxidase, the only one that presents all necessary conditions to deliver electrons directly to the O_2_. However, not all of the energy contained in the electrons will be contained in the ATP, since part of it evolves as heat to maintain the spontaneity of the successive transfers. As the electrons flow through the respiratory chain, they lose their free energy. Part of this energy can be picked up and stored to produce ATP from ADP and inorganic phosphate. The rest of the free energy, which is not taken up for ATP re-synthesis, is released in the form of heat, increasing UCP activity [29, 30].

UCPs are proteins found in the inner mitochondrial membrane that allow proton flow from the intermembrane space to the mitochondrial matrix. However, the return of protons to the mitochondrial matrix does not lead to energy storage in the form of ATP thereby releasing heat. UCP-1, also known as thermogenin, often speeds up the proton return to the mitochondrial matrix so that energy from Krebs cycle, originated from the oxidation of energetic substrates (including lipids), is lost in the form of heat (which can lead to weight loss if this UCP is stimulated) [31].

UCPs can be subdivided into UCP-1, UCP-2 and UCP-3. They differ in their distribution among tissues and possible function. UCP1 is exclusively expressed in brown adipose tissue; UCP3 is expressed in muscle and a number of other tissues; and UCP2 is expressed in a variety of tissues including white adipose tissue (WAT) and is the most highly expressed UCP [32]. These proteins can exert a thermogenic effect and are capable of depleting the proton gradient, but their functions are not yet completely clear [13, 26, 31].

UCP-1 is responsible for lipid oxidation and heat production in brown adipose tissue (abundant in hibernating animals). Human adults have higher levels of white adipose tissue and have UCP-2 and UCP-3, which appears to be related to heat generation. Other UCPs (UCP-4, for instance) are also being investigated [31]. The administration of thyroid hormones leads to respiratory chain uncoupling, which might be explained by an increase in UCPs [33]. Moreover, lipolysis resulting from fasting appears to stimulate UCPs, and the interaction of fatty acids with PPAR seems to increase the expression of UCPs [34].

Supplementation with a CLA mixture or 10,12 CLA in rodents has been shown to induce UCP2 transcription in WAT [32, 35, 36], but whether it plays a role in energy dissipation is unclear. It would appear that CLA interacts with PPARγ, increases CTP-1 and expression of UCP-1 resulting in greater capacity for lipolysis and fat mass weight reduction [28].

## Evidences from experimental animal

According to Gaze et al. [37], the effect of CLA is not the same in all animal models. Rats supplemented with 0.5 % of CLA, for instance, presented a small, but fast (7 days) reduction of adipose tissue, compared to mice [37].

Botelho [8] evaluated the effects of supplementation with CLA on the body composition of healthy Wistar rats supplemented for 3 weeks with CLA at the concentrations of 1 %, 2 % and 4 % on the daily consumption of the diet + control group (2 % linoleic acid). At the end of the period, the groups that were supplemented at a concentration of 2 % and 4 % with CLA presented a greater body fat reduction compared to the control group [8].

Other researchers evaluated coconut oil, maize oil and CLA. In this study 28 rats were allocated to 4 different diets: supplementation with coconut oil, coconut oil and CLA, maize oil and maize oil and CLA. After 28 days, total cholesterol, HDL–c and triglycerides were evaluated. It was found that the triglycerides diminished in the diet supplemented with coconut oil and CLA, and HDL-c diminished with the maize oil diet. The total cholesterol concentrations were lowest in the rats on the coconut oil and CLA diet, but not in the diet with maize oil and CLA. This study suggests that the CLA might diminish adiposity and improve the lipid profile under some conditions [9].

## Evidences from human studies

In recent years, CLA supplementation has also been used in sports, aiming to reduce body fat and possibly improve performance [38]. Furthermore, other supposed benefits include improved lipid profile [39] and/or anti-inflammatory effects [40] that can reduce oxidative stress [41] and ameliorate insulin signaling [42], among others. Collectively, these mechanisms improve body composition and energetic metabolism. Table 1 shows 16 randomized clinical trials (RCT) using CLA as intervention (last 5 years, Pubmed database) on putative benefits. It is possible to see that 9 RCTs, from a total of 16, showed no benefit on aspects related to CLA supplementation. In addition, the studies shown in Table 1, other evidence from human studies are shown below.

**Table 1:** Randomized clinical trials using CLA as intervention on putative benefits

Author	Sampling	Study design	Intervention	Results
BACHMAIR et al., 2015 [55]	Forty-three healthy adults at low to moderate risk of cardiovascular disease	Double-blind, placebo controlled study	Sample received 4 g/day of CLA80:20 or placebo for two weeks	No clear evidence was found for inhibition or activation of platelet function as well as inflammation by CLA80:20 in a low to moderate cardiovascular risk group.
JENKINS et al., 2014a [56]	Thirty-four untrained to moderately trained men	Double-blind, placebo controlled study	Randomly assigned to either a CLA (Clarinol A-80; *n* = 18) or placebo (PLA; sunflower oil; *n* = 16) group Prior to and following 6 weeks of aerobic training (50 % VO_2_peak for 30 min, twice per week) and supplementation (5.63 g of total CLA isomers [of which 2.67 g was 9-*cis*, 11-*trans* and 2.67 g was 10-*trans*, 12-*cis*] or 7.35 g high oleic sunflower oil per day)	Serum triacylglycerol concentrations were lower (p < 0.05) in the CLA than the PLA group. For VO_2_peak and glucose, there were group × time interactions (p < 0.05). However, post-hoc statistical tests did not reveal any differences between the CLA and PLA groups.
JENKINS et al., 2014b [57]	Thirty-four untrained to moderately trained men	Double-blind, placebo controlled study	Randomly assigned to either a CLA (Clarinol A-80; *n* = 17) or placebo (PLA; sunflower oil; *n* = 16) group. Before and after 6 weeks of aerobic training (50 % VO_2_peak for 30 min, twice per week) and supplementation (8 ml CLA or PLA per day), each subject completed an incremental cycle ergometer test, maximal number of sit-ups in 1 min, and the standing long jump	There were no differences between the CLA and PLA groups for the analysis of covariance-adjusted post-test mean values for physical working capacity, sit-ups, or standing long jump. The physical working capacity increased from pre- to post-training in the CLA (*p* = 0.003) and PLA (*p* = 0.003) groups. There were no differences from pre- to post-training for sit-ups and standing long jump in either the CLA or PLA groups. There was no effect of CLA on physical working capacity, maximum number of sit-ups or standing long jump.
ARYAEIAN et al., 2014 [58]	Seventy eight adults with active rheumatoid arthritis	Double-blind clinical trial	Four groups receiving one of the following daily supplement for 3 months; group C: 2.5 g CLAs, group E: 400 mg Vitamin E, group CE: CLAs plus Vitamin E, group P: Placebo. Cytokines, matrix metalloproteinase (MMP-3) and citrullinated antibody (CCP-A)	Co-supplementation CLAs and Vitamin E may be effective in the level of inflammatory markers in rheumatoid arthritis patients.
EFTEKHARI et al., 2014 [59]	Ninety atherosclerotic patients	clinical randomized trial	Patients were classified into 3 groups receiving 3 g/d CLA or 1 920 mg/d ω3 or placebo for 2 months.	Although CLA did not appear to have a significant effect on triglycerides, ω3 supplementation significantly reduced triglycerides level. Consumption of CLA and ω3 supplementation did not significantly affect HDL cholesterol, LDL cholesterol, and total cholesterol.
MOHAMMADZADEH et al., 2013 [60]	Thirty four volunteers patients with rectal cancer	Randomized, double-blind, placebo-controlled pilot	CLA group (*n* = 16), receiving 3 g CLA/d, and placebo group (*n* = 18) receiving placebo capsules (sunflower oil) for 6 weeks	CLA supplementation improved inflammatory factors, MMP-2, and MMP-9 as biomarkers of angiogenesis and tumor invasion. It seems that CLA may provide new complementary treatment by reducing tumor invasion and resistance to cancer treatment in patients with rectal cancer.
PENETO et al., 2013 [61]	Twenty nine healthy adult volunteers (nineteen women and ten men, aged twenty two to thirty six years)	Double-blind clinical trial	CLA depletion was achieved through an 8-week period of restricted dairy fat intake (depletion phase; CLA intake was 5.2 ± 5.8 mg/day), followed by an 8-week period in which individuals consumed 20 g/day of butter naturally enriched with 9-*cis*, 11-*trans* CLA (repletion phase; CLA intake of 1020 ± 167 mg/day)	The intake of a 9-*cis*, 11-*trans* CLA-enriched butter by normal-weight subjects induces beneficial changes in immune modulators associated with sub-clinical inflammation in overweight individuals.
CARVALHO et al., 2013 [62]	Fourteen women diagnosed with metabolic syndrome	Randomized clinical trial, placebo-controlled	Participants received strawberry jam enriched or not with microencapsulated CLA (3 g/day) as a mixture of 38.57 % 9-*cis*, 11-*trans*, and 39.76 % 10-*trans*, 12-*cis* CLA isomers associated with a hypocaloric diet for 90 days	There were no significant effects of CLA on the lipid profile or blood pressure. Mean plasma insulin concentrations were significantly lower in women supplemented with CLA, did not alter the waist circumference, but there was a reduction in body fat mass detected after 30 days, and had a reduced waist circumference
BULUT et al., 2013 [63]	Eighteen sedentary male volunteers	Randomized double blind experiment	Volunteers were randomly divided into CLA and placebo supplementation groups; both groups underwent daily supplementation of either 3 g CLA or 3 g placebo for 30 days and performed exercise on a bicycle ergometer 3 times per week	CLA is not more effective than exercise alone.
LÓPEZ PLAZA, et al., 2013 [64]	Thirty eight volunteers (29w, 9 m)	A prospective, placebo-controlled, randomised double-blind, parallel clinical trial	Volunteers consumed 200 ml/day of skimmed milk with 3 g of CLAs or 3 g olive oil (placebo).	The consumption of skimmed milk enriched with 3 g of a 1:1 mixture of 9-*cis*, 11-*trans* and 10-*trans*, 12-*cis* for 24 weeks led to a decrease in body weight and total fat mass in healthy, overweight subjects who maintained habitual diets and exercise patterns. No adverse effects were observed
ENGBERIN et al. 2012 [65]	Sixty-one healthy volunteers	Double-blind, placebo controlled study	The diets were identical except for 7 % of energy (18.9 g in a diet of 10 MJ/day) that was provided either by oleic acid, by industrial trans fatty acids or by 9-*cis*, 11-*trans* CLA.	The effect of the CLA diet compared with the oleic acid diet was 0.11 mm Hg (95 % confidence interval: −1.27, 1.49) systolic and −0.45 mm Hg (−1.63, 0.73) diastolic. Short-term high intakes of 9-*cis*, 11-*trans* CLA do not affect blood pressure in healthy volunteers.
JOSEPH et al., 2011 [66]	Twenty seven volunteers with overweight, borderline hypercholesterolemic,	Double-blinded, 3-phase crossover trial	Participants consumed under supervision in random order 3.5 g/d of safflower oil (control), a 50:50 mixture of 10-*trans*, 12-*cis*, and 9-*cis*, 11-*trans* CLA:Clarinol G-80, and 9-*cis*, 11-*trans* isomer CLA	Compared with the control treatment, the CLA treatments did not affect changes in body weight, body composition, or blood lipids. In addition, CLA did not affect the β-oxidation rate of fatty acids or induce significant alterations in the safety markers tested.
MICHISHITA et al., 2010 [67]	Forty-one healthy subjects	Single-centre, randomized, double-blind, placebo-controlled trial	Subjects were randomized to receive either placebo or one of three test supplements: amino acid mixture 0.76 g/day; amino acid mixture 1.52 g/day; or amino acid mixture 1.52 g/day coadministered with conjugated linoleic acid 1.6 g/day and exercises for a period of 12 weeks	The results suggest that ingestion of these supplements might enhance the fat-burning effects of exercise.
WANDERS et al., 2010 [68]	Sixty-one healthy women and men	Double-blind, placebo controlled study	It was provided either by oleic acid, by industrial trans fatty acids or by a mixture of 80 % 9-*cis*, 11-*trans* and 20 % 10-*trans*, 12-*cis* CLA.	High intakes of an 80:20 mixture of 9-*cis*, 11-*trans,* and 10-*trans*, 12-*cis* CLA raise the total to HDL cholesterol ratio in healthy volunteers. The effect of CLA may be somewhat less than that of industrial trans fatty acids.
SLUIJS et al., 2010 [69]	Four hundred and one	Double-blind, randomized, placebo-controlled and parallel-group trial	Subjects receive either 4 g CLA/d (2.5 g 9-*cis*, 11-*trans* CLA/d and 0.6 g 10-*trans*, 12-*cis* CLA/d) or placebo supplements for 6 months	There was no effect of 9-*cis*, 11-*trans* CLA supplementation on blood pressure, body composition, insulin resistance, or concentrations of lipid, glucose, and C-reactive protein.
SYVERTSEN et al., 2007 [70]	One hundred and eighteen volunteers	Randomized, double-blind, placebo-controlled trial	Supplementation with either placebo (olive oil) or CLA (Clarinol) for 6 months	CLA does not affect glucose metabolism or insulin sensitivity in a population of overweight or obese volunteers.

Few studies have evaluated changes in body composition with the use of CLA alone or in combination with physical exercise in humans. Blankson et al. [10] showed that CLA may reduce the percentage of fat in humans over a 12-months period, besides increasing the lean mass and not presenting any additional effect at doses above 3.4 g of CLA per day. However since physical training was performed at the same time as the CLA was used, and the levels of exercise were different among the groups, it was not possible to evaluate whether the effect of the body changes was due to the use of CLA, exercise, or the combination of both.

Gaullier et al. [11] performed a 24-month randomized, double-blind placebo–controlled study, during which 6 capsules of gel were administered daily, totaling 4.5 g of CLA. The authors observed that the CLA supplementation for this period in overweight adults is well tolerated, and CLA reduces body fat in overweight humans and can help maintain the initial fat and weight losses over the long term [11].

As to gender, Santos-Zago et al. [20] showed the effect of body fat reduction on healthy, eutrophic women who consumed 3 kg of CLA per day, during 64 days. The results, however, were not significant, since CLA consumption during this period did not alter the women’s body composition [20]. On the other hand, individuals with overweight and obesity, who consumed the amount of 3.4 g of CLA per day for 12 weeks reduced their body fat, as previously shown [10].

The responses to the different CLA isomers do not appear to present differences, although it was found that the 10-*trans*, 12-*cis* isomer increased the concentration of triglycerides and LDL cholesterol in a greater proportion in healthy men compared to the 9-*cis*, 11-*trans* isomer [21]. In a review study, obese men diagnosed with metabolic syndrome used CLA for 4 weeks. The final result was a reduction of the abdominal circumference, however other anthropometric measures did not undergo a relevant change [37].

A randomized, double blind, placebo-controlled study looked at the effects of CLA supplementation on body composition and weight loss for 12 weeks, in individuals with obesity or grade I obesity in the Chinese population. Bioelectric impedance was the method used to evaluate body composition changes during the study. Individuals randomly received 1.7 g of 9-*cis*, 11-*trans* and 10-*trans*, 12-*cis* of CLA (*n* = 30) or placebo (*n* = 33) in 200 ml of sterilized milk twice a day. As a result it was found that the group supplemented with CLA presented a reduction of obesity and/or overweight besides other benefits, without evidence of adverse effects [18].

Kim et al. [43] tested the supplementation of CLA 2.4 g/day CLA mixture (36.9 % of 9-*cis*, 11-*trans* and 37.9 % of 10-*trans*, 12-*cis*) as an antioxidant agent in healthy overweight/obese Korean individuals. Eight weeks of conjugated linoleic acid supplementation has no effect on antioxidant status (plasma total radical-trapping antioxidant potential, lipid peroxidation, lipid-soluble antioxidant vitamin concentration, erythrocyte antioxidant enzyme – superoxide dismutase, catalase, glutathione peroxidase), and leukocyte DNA damage between the CLA, compared to placebo group.

Thirty-seven recreationally-trained women (mean BMI = 25.1 ± 3.4) were randomized for three dietary interventions: 1) a control diet (without changing their usual dietary habits); 2) a high-protein, low-calorie diet supplemented with protein gels (22 g of protein per serving), encapsulated thermogenic (Burn®), multivitamin (Balance®), and CLA (Tone®); and 3) a high-protein diet with isocaloric placebo supplements. After three weeks, women in the supplementation group had their body weight and percentage of body fat (assessed by DEXA and skinfolds) significantly decreased when compared to placebo or control diets. Despite these positive results, it is noteworthy the fact that CLA was used concomitant to other nutritional supplements, and it is thus difficult to assess its effect individually on body adiposity [44].

In another study, 101 moderately obese subjects who lost >8 % of their baseline body weight in a previous study were subsequently assigned to a 1-year double-blind CLA (3.4 g/day) or placebo (olive oil) supplementation regime in combination with a modest hypocaloric diet. The authors found no significant difference in body weight or body fat regain (assessed by DEXA) between the treatments; however, there was a significant increase in the number of leukocytes with CLA supplementation [45].

Individuals with type 2 diabetes mellitus (T2DM) using metformin (30 females and 26 males) were allocated to an eight-week randomized trial and stratified by sex, age and BMI into one of three groups: 1) 3 g CLA/day (3 × 1 g capsules; a 50:50 isomer blend of 9-*cis*, 11-*trans* and 10-*trans*, 12-*cis* CLA) plus 100 IU/day of vitamin E; 2) 3 g CLA/day plus vitamin E placebo; 3) or CLA placebo (soy bean oil) plus vitamin E placebo. By the end of the study there were no significant differences regarding body weight, body composition, glycemic index and inflammatory profile among the three groups; however, there was a trend toward an increase in malondialdehyde levels (a marker of oxidative stress) and decrease in apoB100 (linked to HDL-cholesterol levels) among those receiving CLA [46].

Thirty-five obese postmenopausal women with T2DM were also randomized to receive safflower oil (8 g/day) or CLA (6.4 g CLA isomers/day) in a 36-week randomized crossover trial (two 16-week diet periods separated by a 4-week washout period). DEXA analysis was used for the assessment of body composition. Supplementation with CLA reduced BMI and total adipose mass without changing lean mass; in contrast, safflower oil reduced trunk adipose mass, increased lean mass and significantly lowered fasting glucose. It is suggested that both oils have different effects on body composition in obese women with T2DM who are not also on a weight-loss diet or exercise plan [47].

Finally, a meta-analysis that included 7 clinical assays in the final analysis for the purpose of evaluating the use of CLA during a long time did not show significant results to support changes in the body composition when using CLA for a longer period [48].

## Recommended dose

Most of the studies were a mixture of the two predominant isomers, 9-*cis*, 11-*trans* and 10-*trans*, 12-*cis*, in equal proportions. The daily doses of CLA varied from 3 to 6 g/day, according to several studies and these doses appear safe [49]. Although some studies indicate that doses above 3.4 g/day would not have any additional effect, they suggest that there is a very great variation compared to the results due to different doses, type of isomer, and evaluation of the body composition, which makes it difficult to compare different studies [10, 18].

## Adverse effects

Despite the positive effects of CLA supplementation on some health-related parameters, there are a few reports of possible adverse effects, mainly in rats and due to the 10*-trans* and 12-*cis* isomer. In the animal models pro-carcinogenic effects and of increased production of prostaglandins attributed to CLA 10-*trans* and 12-*cis* have been identified [50].

Other negative effect may be due to the increase in the lipid oxidation products (isoprostanes), besides the diminished leptin and greater probability of developing insulin resistance [51]. Studies also show undesirable effects in human beings as increased levels of triglycerides and LDL-cholesterol and reduction of the HDL levels, suggesting a negative alteration in the serum lipids profile [52]. Obese individuals also presented negative alterations in the glucose metabolism with insulin resistance in some studies [53, 54].

## Conclusions

Despite studies on CLA supplementation for the purpose of investigating changes in body composition and other benefits, both in animals and in humans, they are very discordant. The capacity of CLA to alter the body composition positively by reducing the fat mass was proved in experimental models, and, in some studies on human beings. In fact, few studies have evaluated the use of CLA alone or in combination with physical exercise in humans, regarding changes in body composition. Therefore, the clinical evidence appears to be insufficient and not unanimous regarding the effects on body fat reduction and major side effects have already been described.

In this sense, the consumption of foods naturally enriched with CLA (and not from supplementation) during lifetime would be an alternative to reduce increased adiposity. Besides, it could reduce de risk of other diseases associated with obesity, since they would ensure the beneficial effects on body composition and would not add effects that are adverse to health.
